# Pain modulation by self-generated expectations

**DOI:** 10.1038/s41598-025-17276-8

**Published:** 2025-08-27

**Authors:** Carla Mourkojannis, Maren-Isabel Wolf, Christoph A. Wittkamp, Michael Rose

**Affiliations:** Department of Systems Neuroscience, University Medical Center Hamburg‑Eppendorf, Martinistr. 52, Building W34, 20248 Hamburg, Germany

**Keywords:** Human behaviour, Neuroscience

## Abstract

Pain perception is an individual and complex process, influenced by biological, social, and psychological factors via top-down modulatory pathways. One factor which plays an important role in pain perception is expectations, which have been popularly examined in placebo and Hyperalgesia studies, showing that pain perception can be modulated by manipulating expectations of treatments, pain stimuli or outcome. This preregistered EEG study provides compelling evidence for the high efficacy of self-generated expectation in modulating pain perception in direct comparison to externally induced expectation in a typical placebo paradigm. 42 participants were asked to generate expectations based on visual cues towards a subsequent pain stimulus and either lighten, enhance, or not actively influence their pain expectation. The behavioural results showed that pain perception was significantly altered by self-generated expectations, and that all three conditions differed significantly from each other regarding the reported pain. Notably, self-regulation produced more pronounced effects than externally induced expectations in direct comparison to the results of a previous placebo interventions using the identical experimental layout, indicating that deception is unnecessary for effective pain modulation. EEG analyses revealed differential neural processing among conditions, particularly within gamma-band activity linked to pain perception, underscoring the influence of self-generated expectations on neural responses. These findings provide evidence that even expectations created on a voluntary basis directly influence pain perception, and that manipulating strategies used in placebo studies such as conditioning, verbal instructions and observational learning may not be necessary to evoke the described effects.

## Introduction

Nociceptive input can lead to a wide range of pain sensations, with expectations playing a critical role in the modulation of pain^1^. Positive expectations can result in hypoalgesia (the placebo effect), whereas negative expectations may enhance the perceived intensity of pain (the Hyperalgesia effect). Both well-known effects are typically experimentally initiated by verbal instructions and a conditioning phase. During this process, volunteers or patients are usually explicitly told that there is an external active treatment, although it is only a sham treatment. In general, this intervention is highly effective^2–4^. A meta-analysis taking into consideration 20 studies with a total of 603 healthy subjects came to the result that in 17 of the 20 studies and in the combined sample placebo manipulations caused significant behavioural reductions on pain ratings^5^. However, placebo interventions only offer limited clinical applications due to the deceptive nature of this treatment, warranting the need for other approaches.

Previously, it has been shown that active engagement in the treatment process (or agency) increases placebo analgesia^6,7^indicating that the feeling of control is an important modulator during pain processing. The conveyance of the feeling of control and self-regulatory strategies is also an essential aspect of pain-related psychotherapy, aiming to alter patients’ explicit expectations of clinically relevant events and stimuli through reframing, cognitive restructuring, or other techniques^8^. The success of this approaches demonstrated that the feeling of control is an important modulator of pain processing.

Further, placebo effects can be induced by honestly prescribed open-label placebos (OLP), thus complying with principles of medical ethics. Here, volunteers or patients are explicitly informed about the sham nature of the treatment. The results of open-label placebo studies question whether deception is a necessary characteristic of placebo effects^9^. A study directly comparing OLP and deceptive placebo showed that deception may not be necessary for effective placebo treatments and that OLP approaches may offer an important opportunity for the management of pain^10^. Despite their heterogeneity, these studies suggest that OLP effects do not merely reflect a reporting bias and offer the potential of ethical open-label and dose-extending placebos to improve health outcomes in patients and reduce side effects^11^. Mainly, these OLP studies with informed volunteers or patients focused on the placebo effect, i.e. a reduction in felt pain. It is an open question whether an increase in the pain sensation can be achieved by this kind of manipulation.

In contrast to OLP settings, studies on self-regulation of nociceptive input focus on the self-efficacy and control of the volunteers and showed a clear modulation of pain processing and the involvement of a distinct neural network responsible for this modulatory influence^12^. Here, a direct comparison to a placebo intervention would is missing.

Both OLP and perceived control can be effective for a modulation of pain sensation, therefore a combination of the key elements from both approaches could enhance the ability of top-down modulatory influence on pain processing.

The aim of the present study was to give the volunteers open information about the clear influence of expectations on pain processing and to enhance control by giving them the opportunity to self-generate positive and negative expectations about an upcoming nociceptive stimulus based on the information of visual cues. We aimed to directly compare externally generated expectations within an established placebo/Hyperalgesia paradigm with self-generated expectations within the identical paradigm to compare the efficacy of both approaches.

In a previous study, we investigated the generation of positive and negative expectations, and the formation and integration of expectations into pain perception, using a novel paradigm that allowed the manipulation of expectations on a trial-by-trial basis^13^. We presented visual cues to externally induce expectations (positive, negative, or neutral expectations) by instructing the participants that they would be given real-time visual feedback on their current pain sensitivity based on their EEG activity using a Brain-Computer Interface (BCI). The feedback would indicate one of three different brain states: either a state of high pain sensitivity (red cue/Hyperalgesia condition/negative expectation), low pain sensitivity (green cue/placebo condition/positive expectation), or that the BCI algorithm would not make any prediction (yellow cue/control condition/neutral expectation). In fact, the visual cues were not related to any brain activity but were only used to produce the corresponding expectations and varied from trial to trial. Behavioral results clearly demonstrated enhanced pain perception under the Hyperalgesia condition and reduced pain ratings for the placebo condition. The presentation of the cues was followed by an anticipatory period in which the different expectations emerged. This was followed by the presentation of a painful heat stimulus. Results of the neural data revealed substantially different neural representations between the anticipatory and the actual pain period and an involvement of high frequency oscillatory activity (gamma band) measured with EEG. A follow-up study further showed the long-term stability of the effects across one week^14^.

In the present study, we used the identical experimental layout as in the previous study but clearly informed the volunteers about the nature of the visual cues and instructed them to use the cues to voluntarily generate corresponding expectations. We hypothesise that the Hyperalgesia Condition result in increased pain and expectation ratings compared to the Control Condition and vice versa for the Hypoalgesia Condition. Due to the factor control we hypothesize to observe larger effects in the present study compared to the previous study. As a proxy for the influence of these expectations on the pain processing, we measured time-frequency decomposed EEG. Previous studies demonstrated the relation of in particular high frequency oscillatory activity in the gamma band (around 40 Hz) and the perceived pain intensity^15,16^. It has been previously demonstrated that gamma band activity during pain processing is modulated as a consequence of cognitive demands and -in particular- by control or agency^7^predictions in visual processing^17^ as well as during expectations on pain^13^. In accord with these results, we hypothesize a difference in gamma band activity between all three conditions based on the different pain processing.

### Participants

A total of 42 healthy adults participated in the experiment (28 female, 14 male, age M = 24.36 years, SD = 3.19 years). Participants were recruited via a local online job market (stellenwerk.de/hamburg) and advertising at the University of Hamburg. The participants gave their written informed consent before the beginning of the experiment and were compensated with 15€/h. All participants were 18–35 years old, right-handed, non-smokers, and reported normal or corrected-to-normal vision, no red-green-colorblindness, no chronic or acute illness or pain, no neurological or psychiatric disorders, no pregnancy, no regular use of medication (except contraception) or recent use of pain killers, no consumption of illegal drugs, and also no participation in another medication or pain associated experimental study in the past six months. The study was preregistered (German Clinical Trials Register; ID: DRKS00026174) and approved by the local ethics committee (Medical ethic committee Hamburg: PV7170). We ensure that all research was performed in accordance with relevant guidelines and regulations.

### Procedure

**Fig. 1:**
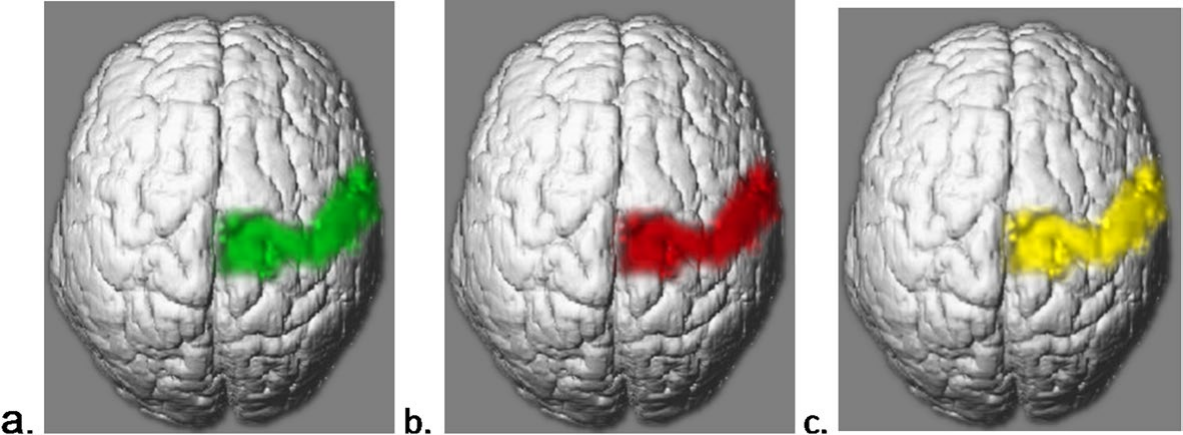
shows the three different visual cues, which have been shown during the experiment to indicate the current condition. (**a**) represents the Hypoalgesia condition, in which pparticipants were instructed to generate the expectation of a weaker pain experience. (**b**) the Hyperalgesia condition in which pparticipants were instructed to generate the expectation of a stronger pain experience and (**c**) the control condition.

The experimenter introduced the participant to the topic and procedure of the experiment by reading a formulated introduction which included information about the Hypoalgesia effect and the proven influence of expectations on pain perception. Subsequently, the experimenter familiarized the participant with the three conditions of the experimental trials, which were indicated by visual cues: The Hypoalgesia Condition: Participants were instructed to generate the expectation of a weaker pain experience (see Fig. 1a), the Hyperalgesia Condition: Participants were instructed to generate the expectation of a stronger pain experience (see Fig. 1b) and third, the Control Condition: Participants were instructed to generate no specific expectation of the following pain experience (see Fig. 1c). The participants were further informed that the actual pain stimulus would be independent of the visual cue and would vary from trial to trial in its intensity. No distinct self-regulatory strategy was given to the participants, but only the instruction to try to expect more or less pain.

### Calibration phase

During the calibration phase, pain stimuli were inflicted via short painful thermal stimuli with a duration of 4 s at target temperature to the proximal volar mid-forearm. To deliver heat stimuli, a PATHWAY CHEPS (Contact Heat-Evoked Potential Simulator) thermode was used (https://www.medoc-web.com/pathway-model-cheps). Its rapid heating rate amounts to 70 °C/sec and its cooling rate to 40 °C/sec. Furthermore, pain stimuli in the range of 30 °C to 55 °C in less than 300 ms can be delivered. The baseline temperature was set to 32 °C and the rise and fall rate was set to 70 °C/sec during the calibration phase and subsequent phases. Individual temperatures were determined on a visual analogue scale (VAS) from 0 (“no pain”) to 100 (“unbearable pain”) by using a stepwise procedure. Unbearable pain was defined as the maximum of pain the participants were willing to endure during the experiment. Three different values (VAS30, VAS60 and VAS70) were assessed for each participating subject. The final target temperature of VAS60, referring to a pain stimulus with average pain intensity, was calculated using linear regression.

### Experimental phase

**Fig. 2:**
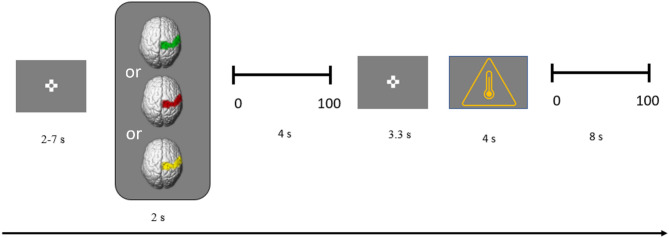
Chronological sequence of one trial, showing the ITI before the presentation of a visual cue, followed by the expectation rating, the expectation phase, the provision of the pain stimulus and the pain rating.

Each trial began with the presentation of one cue stimulus according to one of the three conditions (red, yellow, or green visual cue) for two seconds to create an expectation of the pain intensity in the participant. Next, the participant should rate his/her expectation of the following pain experience on a visual analogue scale (VAS) from 0 (“no pain”) to 100 (“unbearable pain”) by using the arrow keys of the keyboard to locate the cursor and confirming his/her expectation with “return” in less than 4 s while the visual cue continued to be presented on screen. The anticipation phase followed with a fixation cross being shown on the screen for 3.3 s, just before the pain stimulus was induced with a duration of 4 s. Finally, another VAS was presented for 8 s to rate the perceived pain intensity. Between trials, there was a fully randomized inter trial interval (ITI) of between 2 and 7 s. The trial run is graphically represented in Fig. 2. Pain stimuli were of identical intensity in all trials, corresponding to their individual value of VAS60 which was determined during the calibration phase. Participants were not aware that they were receiving the same pain stimulus intensity in all trials during the experiment. To familiarize the participants with the task, six test trials that were identical to the experimental trials preceded the first experimental block, consisting of two trials of each condition in random order.

#### Graphical representation of one trial

The experimental phase consisted of three different blocks, including 30 trials and taking 12–20 min each. Each block started with the application of one heat pain stimulus corresponding to VAS60 to ensure that there were no technical issues. After the participant confirmed the reception of the heat stimulus, the trials started. The trials were in pseudorandom order with no more than two direct repetitions of the same condition. After each block, the participants had the opportunity to take a small break and the position of the thermode was changed to being either proximal or distal located on the volar mid-forearm (block 1: proximal, block 2: distal, block 3: proximal).

After the third block was finished, the participant was asked to answer the following questions: ”Did you notice anything particular?”, “Did you have the impression that your pain perception was influenced by your expectations?”, “Have you noticed any differences in the intensity of the pain stimuli?”, and “Did you use any strategies to modulate your expectations?”.

### Data acquisition

#### EEG data

For the acquisition of the EEG data, a 64-channel actiCAP and the BrainVision Recorder (BrainProducts, Gilching, Germany) were used. The experiment was conducted inside an electrically shielded laboratory. The cap was composed of 64 active Ag/AgCl electrodes of which 62 were arranged according to the extended 10/20 System whereas two electrodes recorded a bipolar horizontal electrooculogram (HEOG). FCz functioned as reference and Pz as ground electrode. Two BrainAmp amplifiers with 32 channels each (BrainProducts, Gilching, Germany) were connected to the cap. The sampling rate to record the data was 500 Hz with an amplitude resolution of 0.1 µV. The EEG-Data was filtered online with a low cut-off filter with a time constant of 10 s and a high cut-off at 1,000 Hz. The electrode skin impedance did not exceed 20 kΩ.

#### Preprocessing

To preprocess the EEG data, the Fieldtrip toolbox for Matlab (Oostenveld at al. 2011) was used. The data was segmented into trials from cue presentation (1,000 ms prior) to the end of pain stimulation (3500 ms after cue onset). Subsequently, the segmented data was filtered (low-pass filter at 150 Hz, high-pass filter at 0.5 Hz) and split into low- and high-frequency data (34 Hz low-pass filter and 16 Hz high-pass filter, respectively). It was then processed in parallel to secure maximal sensitivity in detecting and removing artifacts. In addition, all single trials were visually inspected and removed for both high- and low-frequency data in case of excessive artifacts (a mean of 11 trials were excluded, SD = 2.4, range across participants 4–26). Next, both subsets underwent independent component analysis (ICA) throughout a logistic infomax algorithm. Components like blinks, eye- and head movement, or muscle activity were visually identified via time course, spectrum, and topography and could thus be discarded (a mean of 4 components were excluded, SD = 0.9, range across participants 3–11). Both subsets were re-referenced to the average of all EEG channels and the original reference electrode (FCz) was regained.

### Data analysis

#### Behavioral data

Two different behavioral data were acquired: the Expectation Ratings that are given prior to the pain stimulus and after visual cue and Pain Rating that are acquired after the pain stimulus. Both are measured under three different Conditions (red, green or yellow cue). The effects of self-generated expectations on Expectation and Pain ratings under the different Conditions were analysed using a linear mixed-effects model (LME) that incorporated the fixed effect of condition and the random effect of subject, based on single-trial data, followed by a subsequent ANOVA on the fitted model to examine the significance of the fixed effects. In detail, for the analyses within the present experiment the dependent variable ‘Expectation Rating’ (before the pain stimulus) or the actual ‘Pain Rating’ (after the pain stimulus) is modelled as a function of `Condition`. To test an effect across time we also modelled both analyses with the additional factor ‘block’.

For the comparison between the experiments both dependent variables (‘Expectation’ or ‘Pain Rating’) are modelled as a function of Condition` and `Experiment` (the present study and the previous experiment), and their interaction (`Condition * Experiment`). All models include a random intercept for each subject.

Based on this analysis, condition-specific comparisons were computed and corrected using Bonferroni corrections.

For a direct comparison with the original placebo study, the mean pain level of each participant was subtracted from each condition and the pain ratings were compared using a LME that incorporated the fixed effect of condition and experiment based on single-trial data, followed by a subsequent ANOVA and Bonferroni adjusted comparisons.

#### EEG data

The three different conditions were compared using a univariate repeated-measures ANOVA on oscillatory activity during the pain processing (Fieldtrip toolbox). The analysis was restricted to the time period of pain (0: 4s from pain onset) to test for differences of pain processing in relation to the different conditions and from 4 to 128 Hz. Power differences were statistically tested via nonparametric cluster-based permutation F-test as implemented in the Fieldtrip toolbox (cluster threshold: *p* = .05, number of randomizations: 2000).

## Results

### Behavioural data

**Fig. 3:**
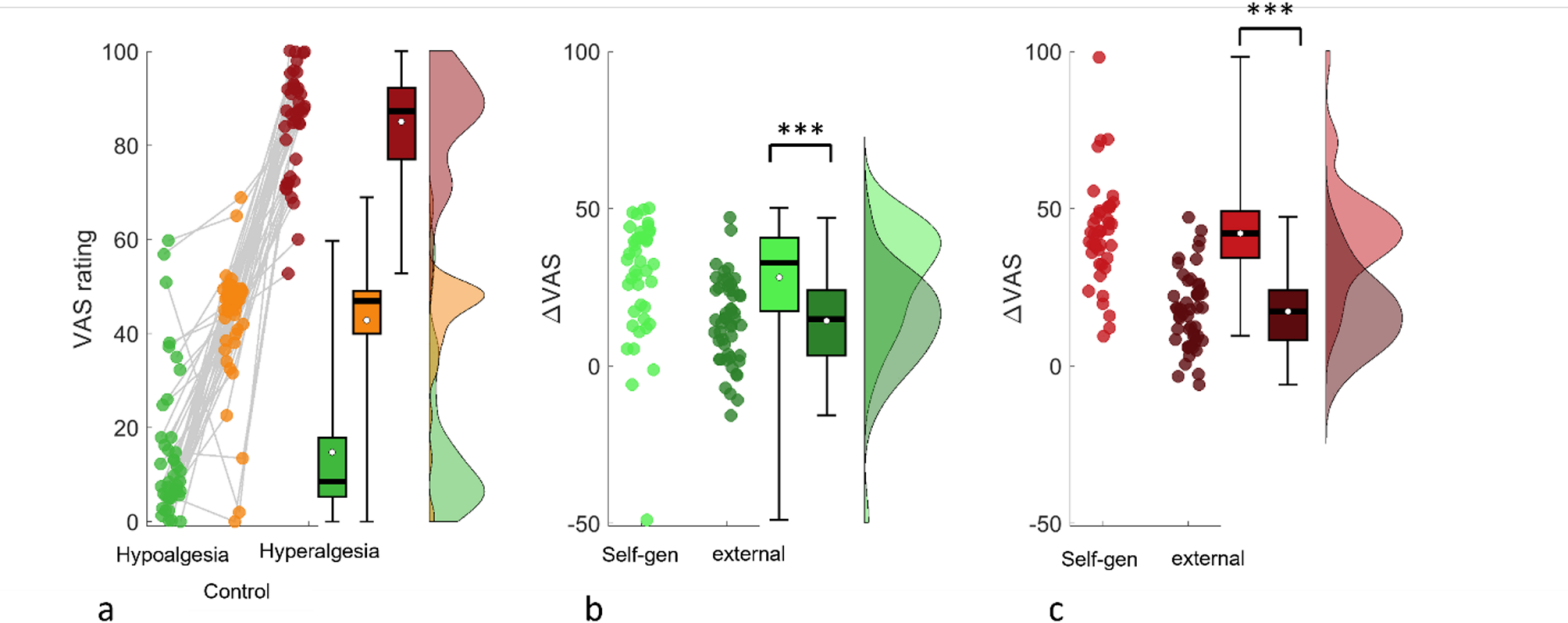
(**a**) *Left*: Raincloud plots of expectation ratings per condition from the study using self-generated expectation. Comparison of the different conditions between experiments for the Hypoalgesia conditions and **c**) the Hyperalgesia conditions (both plotted as a difference to the control condition). Larger effects were observed for positive and negative expectations. ***p < .001. Error bars represent the corrected standard error of the mean using the Cousineau-Morey method^18,19^.

Volunteers rated their expectations regarding the pain intensity of the subsequent thermal stimulus. These expectation ratings were significantly modulated by the condition cues, as evidenced by a linear mixed effect analysis (LME ANOVA) (Condition: F(2,3777) = 239.1, *p* < .001). Bonferroni corrected comparisons showed that expectation ratings were higher in the self-generated hyperalgesia condition (M = 84.93, SE = 1.7) compared to the control condition (M = 42.79, SE = 2.0) and to the hypoalgesia condition (M = 14.76, SD = 2.3) and between hypoalgesia and hyperalgesia condition (all *p* < .001, see Fig. 3).

**Fig. 4:**
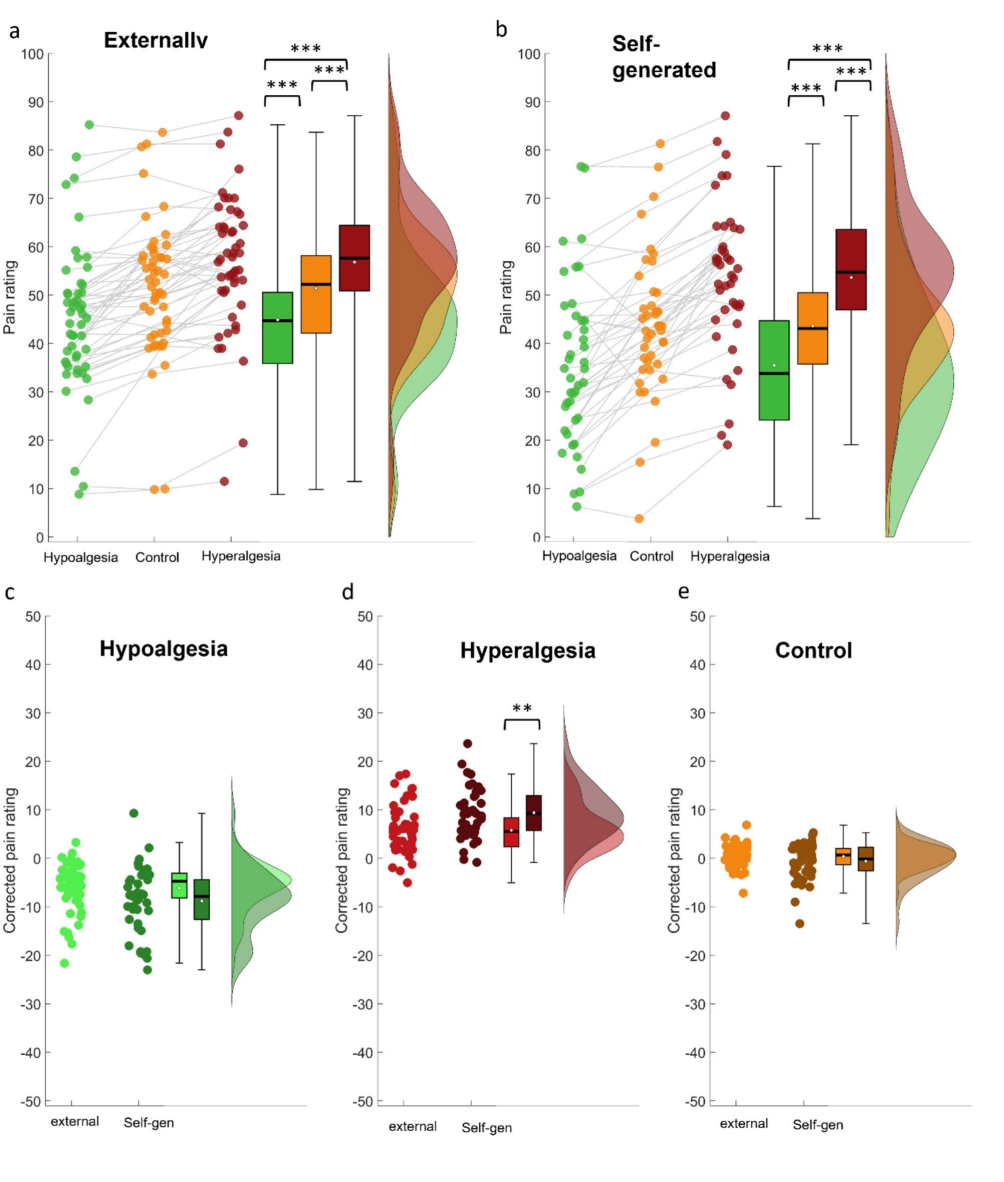
(**a**) *Left*: Raincloud plots of pain ratings per condition from the study using external expectation induction (Hypoalgesia, Hyperalgesia and control). Individual dots denote the average rating of a subject per condition, with grey lines connecting the ratings of the same subject over conditions. Black thick line = Median. White dot = Mean. (**b**) Results of the present study using the self generated expectations within the identical experimental layout. (**c**)-(**e**) Comparison of the different conditions between experiments corrected for the mean pain level in each subject. Larger effects were observed for positive and negative expectations in the self-generated task, while ratings were similar in the control condition. *p < .05. **p < .01. ***p < .001. Error bars represent the corrected standard error of the mean using the Cousineau-Morey method^18,19^.

For the pain ratings after the thermal stimulus, the analysis revealed a clear influence of these expectations on pain processing as demonstrated by a LME analysis and the reliable differences between the conditions (Condition: F(2,3777) = 68.3, *p* < .001). Bonferroni-holm corrected contrasts showed that participants rated pain stimuli in the hyperalgesia condition (M = 53.64, SE = 2.3) higher than in the control condition (M = 43.64, SE = 2.3) and in turn in the control condition higher than in the hypoalgesia condition (M = 35.45, SE = 2.6), and a reliable difference between hypoalgesia and hyperalgesia condition (all *p* < .001, ,see Fig. 4). These analyses indicated that self-generated expectations had large effects on the expectation and perception of painful stimuli. To test for the effect of habituation on Pain and Expectation ratings we included the factor ‘block’ (3 blocks) in the LME model. For the Pain Ratings a clear habituation effect was revealed (block: F(2,3771) = 3.8, *p* < .05) but no interaction with the different conditions, indicating that the self-generated expectations are stable over time regardless of the habituation effect. This is supported by the fact that the Expectation ratings did not show an effect of block (F(2,3771) = 0.1, *p* = .90).

In the next step, we directly compared the self-generated pain modulation effects observed in this study to those externally generated in a former study using the identical experimental design. For a better comparability, the mean pain level of each participant was subtracted from each condition. The analysis of the pain ratings revealed reliable differences between the conditions across both experiments (Condition: F(2,8274) = 332.8, *p* < .001) and a general difference between the experiments (Experiment: F(2,8274) = 26.9, *p* < .001). A reliable interaction between Condition and Experiment (F(2,8274) = 21.5, *p* < .001) indicated a differential effect between the conditions. Bonferroni corrected contrasts showed a significant difference between the pain reduction conditions in the two experiments ( t(2759) = 3.8, *p* < .001), as well as between the pain increase conditions (t(2749) = 5.0, *p* < .001), but not between the control condition in both experiments (t(2766) = 1.3, n.s).

Thus, reported hypoalgesia and hyperalgesia effects were not only comparable to those deceptively generated, but even larger.

Also, the modulation of expectations ratings were clearly larger in the group that self-generated the expectations. The analysis of the expectation ratings revealed reliable differences between the conditions across both experiments (Condition: F(2,8274) = 5706, *p* < .001) and a general difference between the experiments (Experiment: F(2,8274) = 72.5, *p* < .001). The expectation ratings in the self-generated group may be affected by the instruction and a comparison is limited between groups. However, the larger effects in the self-generated group showed that the believe in the expectations was higher in the self-generated group.

### EEG

**Fig. 5:**
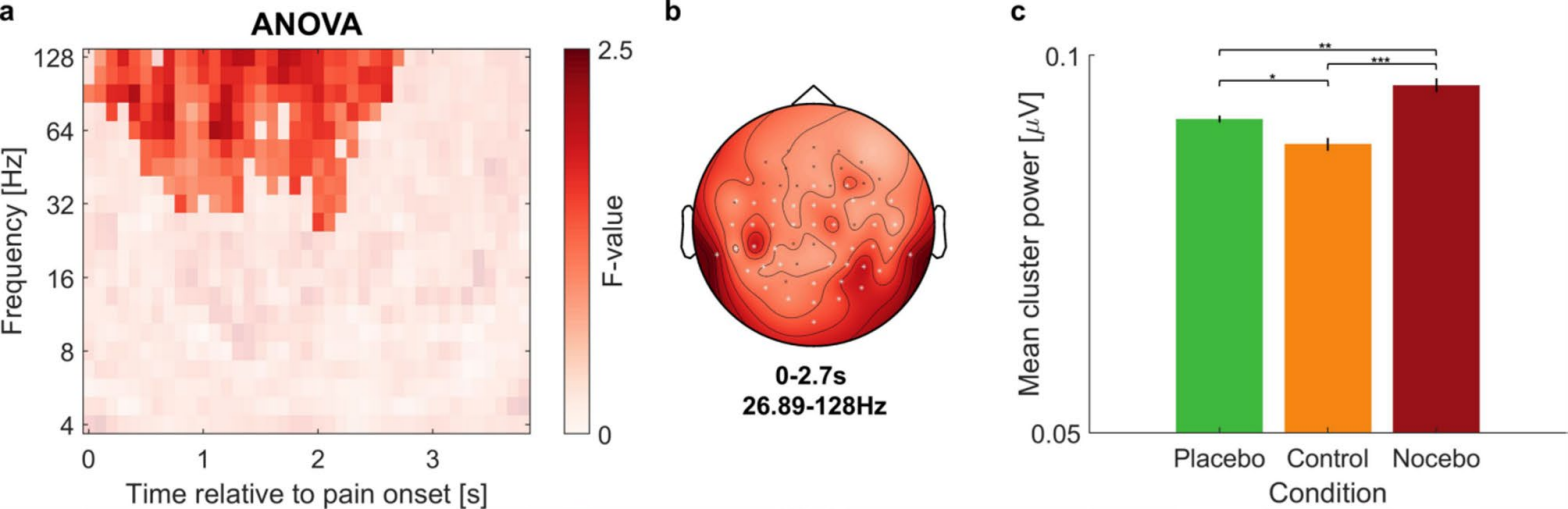
Differences in EEG oscillatory activity between all three conditions. (**a**) Time-frequency plot of *F*-values comparing all three conditions during the pain phase, averaged over all electrodes within the cluster. Non-significant time-frequency points are masked. (**b**) The topography of gamma effects over all frequencies and time points included in the cluster. Cluster channels are highlighted with a white star. (**c**) Raw power within the cluster was extracted per condition for each subject. A rmANOVA showed that gamma activity was highest in the Hyperalgesia condition, followed by the Hypoalgesia condition, and gamma power was lowest in the control condition. Error bars represent the corrected standard error of the mean using the Cousineau-Morey method^18,19^. *p < .05. **p < .01. ***p < .001.

Next, we assessed whether self-generated expectations led to differences in EEG oscillatory activity during the processing of the pain stimuli as an objective measure for differential processing. Cluster-based permutation tests revealed significant differences in gamma band activity between the three conditions, mainly in posterior electrodes across a time range of about 2.7s (*p* = .017, 26.89–128 Hz; see Fig. 5). To further explore this effect, we extracted the mean power within the cluster per condition for each participant and conducted a rmANOVA comparing the values between the three conditions. There was a significant effect of condition (F(1.37,56.35) = 16.84, *p* < .001, η2p = 0.29). Power within the cluster was higher in the Hyperalgesia (M = 0.096, SD = 0.082) compared to the Hypoalgesia condition (M = 0.092, SD = 0.078; pholm = 0.003) and control condition (M = 0.088, SD = 0.075; pholm < 0.001), and in the Hypoalgesia condition higher compared to the control condition (pholm = 0.016). This suggests that the expectation modulation indeed led to differences in the neural processing of the pain stimuli in relation to the different cues.

### Reported strategies

Regarding the subjective efficacy of the procedure, most participants stated that their expectations had at least partly modulated their pain perception (71.43%). As we did not instruct a specific strategy, we were also interested in which strategies were used. A variety of strategies to maintain and implement expectations was reported, e.g., internal verbalization (21.43%), imagination (14.29%), and distraction (only for the green cue; 9,52%), while some subjects stated using no specific strategy (54.76%). Even though the group size was small for some strategies, we tested for difference between the reported strategies in the success for positive or negative expectations and observed no reliable difference (positive modulation: F(3) = 0.72, *p* = .55 ,negative modulation: F(3) = 0.78, *p* = .51).

## Discussion

The results of the present study clearly supported the high efficacy of self-regulatory processes in direct comparison to a deceptive placebo intervention. The expectation and pain ratings in the present study differed between all three conditions, although the actual thermal stimulus was identical in all trials. The EEG results demonstrated a differential processing between all three conditions and therefore argue for a modulated pain processing based on self-generated expectations. The pain modulation by self-generated expectations was consistently higher than the externally induced expectations using the identical paradigm, demonstrating that deception is not necessary for a successful pain modulation. In the previous placebo study, clear behavioural effects were observed with a large effects size, but the self-generated expectations in the present study were even more effective for the pain modulation in both directions. This is in contrast with the results observed in the open-label placebo studies that reported no difference between placebo and open-label placebo or a reduced effect in the open-label condition^9,10,20,21^.

However, the effects from this novel type of modulation are consistent with findings from previous studies showing that self-regulation of pain perception can effectively affect reported pain^12,22^ and that self-regulatory strategies are integral to cognitive behavioural treatments of chronic pain^8^. Findings revealed that a distinct neural fronto-striatal pathway connecting nucleus accumbens and ventromedial prefrontal cortex mediates the effects of cognitive self-regulation of pain^12^. Self-regulatory strategies establish the feeling of control, and it was shown that this factor is a strong modulator of pain processing^6,7^ Further, it was found that even in the context of open-label placebos, control can enhance the effectiveness of the sham treatment^23^. The feeling of control affects many other cognitive aspects like reward processing and anxiety modulation^11^but it is unclear which factor really affects the pain processing.

Although the modulation in both directions was higher using self-generated expectations than the externally given information, interestingly the increase of the pain sensation was more pronounced than the decrease of pain. This tendency was also observed in previous studies^13,14,24^indicating probably an evolutionary relevant tendency to expect harmful events and therefore a higher priority of these signals^25^.

Self-regulation is a large field, and it has been shown that not all forms of cognitive modulation are equal, and that different forms of psychological modulation influence pain and emotion via distinct systems^26–28^.

Self-generated thoughts for example appeared to be difficult to influence pain substantially, while some forms of cognitive regulation, e.g., distraction and placebo manipulations, may regulate pain at very early stages of processing in the spinal cord in some cases^29,30^.

A wide range of different strategies can be used to influence pain processing like diversion of attention, reappraisal, imagery, and control^22^. In clinical practice, cognitive strategies are widely used to reduce pain sensation, as they are key mechanisms of cognitive behavioral therapy and mindfulness-based interventions^31^. Specifically, psychological interventions for chronic pain in older adults have been found to lead to small but consistent effects concerning pain reduction, decreased catastrophizing beliefs, and improved self-efficacy in pain management^32^. However, it remains unclear whether any specific cognitive strategy is more effective than others. While some studies did not report significant differences^23^others found differences in the effectiveness in the observed strategies^22^. Also, on an individual level, the effectiveness of specific strategies remains ambiguous: Whilst Forys and Dahlquist^33^ reported that patients with chronic pain find specific strategies more effective depending on their personal coping mechanisms, Lawrence et al. came to the conclusion that the average self-reported ability to modulate pain is similar across different strategies and reported that most participants did not perceive one strategy superior to another^34^. This is in accordance with the results of the present study in which no specific strategy was instructed and no differences between the reported strategies were observed. Further, no specific training was provided for the volunteers. Nevertheless, we observed a clear modulatory influence on the perceived pain that is significantly higher than the effects observed in the previous study using the same experimental layout but with specific placebo instructions. Therefore, self-generated expectations showed a higher effect on the pain perception than the expectations evoked by the pseudo-treatment of the placebo interventions.

An alternative interpretation of the behavioural results of the pain rating is that self-generated expectations do not directly impact pain but rather induce a sort of ‘‘decision bias” related to the instructions. However, the EEG results argue against this hypothesis since the differential activity within the gamma band during pain processing indicated a modified neural processing that result in the perceived pain. The gamma band activity during pain processing has been previously shown to reflect felt pain^15,17,35^ and modulatory influences on pain processing^13,35^. In particular, the relevance of gamma band oscillatory activity has been shown to reflect top-down factors like by control or agency^7^predictions in visual processing^17^ as well as expectations on pain^13^. Therefore, the differential processing observed by the gamma band activity clearly argues for a differential processing of the thermal stimulus and not just a response bias during the rating process. Further, a “decision bias” related to the given instruction could also affect the ratings within a typical placebo study and therefore may partly affect both interventions.

The intervention of consciously modulating expectations goes beyond OLPs by illustrating that participants may voluntarily enhance the efficacy of inert treatments, despite being aware that the condition cues did not indicate actual changes in stimulation. Differently to OLPs, the present approach heightens feelings of control beneficiary for the processing of pain. Another important difference is that participants who benefit from OLPs often do not report positive expectations concerning the procedure, suggesting that more unconscious and embodied processes may mediate those effects^36^. In contrast, the participants of the present study rated their expectations consistent with the condition cues, indicating a more conscious process with a clear effect on the sensation of pain.

Practical implications of these findings include the potential to educate patients about the influence they may have over their own expectations related to treatments and perceptions. This may also enhance the perceived control that patients have over their own treatment, which could facilitate positive expectations and the placebo effect.

In summary, the present results clearly showed that expectations created on a voluntary basis directly influence pain perception and that this effect is even larger than placebo interventions within an identical experimental design. In can be concluded that a manipulation used in placebo studies such as conditioning, verbal instructions and observational learning may not be necessary to evoke the described effects. This observation underlines the high potential of self-regulatory strategies in pain management.

## Data Availability

Data will be provided at https://www.fdr.uni-hamburg.de/record/17805.
